# Biodegradation of 17β-estradiol by Bacterial Co-culture Isolated from Manure

**DOI:** 10.1038/s41598-018-22169-0

**Published:** 2018-02-28

**Authors:** Mingtang Li, Xingmin Zhao, Xiufang Zhang, Di Wu, Su Leng

**Affiliations:** College of Resource and Environmental Science, Key Laboratory of Sustainable Utilization of Soil Resources in the Commodity Grain Bases in Jilin Province, Jilin Agricultural University, Changchun, 130118 PR China

## Abstract

Animal wastes are potential sources of natural and steroidal estrogen hormones into the environment. These hormones can be removed by microorganisms with induced enzymes. Two strains of 17β-estradiol-degrading bacteria (LM1 and LY1) were isolated from animal wastes. Based on biochemical characteristics and 16 S rDNA gene sequences, we identified strains LM1 and LY1 as belonging to the genus of *Acinetobacter* and *Pseudomonas*, respectively. Bacterial co-culture containing LM1 and LY1 bacterial strains could rapidly remove approximately 98% of E2 (5 mg L^−1^) within 7 days. However, strains LM1 and LY1 degraded 77% and 68% of E2 when they were incubated alone, respectively. More than 90% of 17β-estradiol (E2, ≤ 20 mg L^−1^) could be removed by bacterial co-culture. Low C/N ratio (1:35) was more suitable for bacterial growth and E2 degradation. The optimal pH for bacterial co-culture to degrade E2 ranged from 7.00 to 9.00. Coexisting sodium acetate, glucose and sodium citrate decreased E2 degradation in the first 4 days, but more E2 was removed when they were depleted. The growth of the bacterial co-culture was not significantly decreased by Ni, Pb, Cd or Cu at or below 0.8, 1.2, 1.6 or 0.8 mg L^−1^, respectively. These data highlight the usefulness of bacterial co-culture in the bioremediation of estrogen-contaminated environments.

## Introduction

Steroid estrogens are frequently found in water and soil. Recently, steroids contamination becomes a significant problem in the worldwide due to their unfavorable effects on reproductive, neurobehavioral and other endocrine-mediated functions of humans and animals^1–3^. Among them, estrone (E1), 17β-estradiol (E2), estriol (E3) and 17α-ethinylestradiol have been the focus of many studies because they have been frequently detected in water, manure composts, and soil^4–9^. Recent studies have shown that natural estrogen from animal wastes of animal feed operation sites can cause air, water and soil pollution, thereby posing risks to organisms^5,6,10^. Therefore, removal of estrogens from animal waste can help to preserve the ecosystem.

Microorganisms play an important role in the degradation of natural estrogens. Many estrogen-degrading bacteria have been isolated from waste water, soil or animal wastes^11–15^. However, only a few bacterial strains can completely degrade estrogen into CO_2_ and H_2_O as final products. For example, *Comamonas testosteroni* can transform steroids to CO_2_ and H_2_O through a complex catabolic pathway that involves a set of steroid-inducing enzymes such as 3α-hydroxysteroid dehydrogenase/carbonyl reductase and 3,17β-hydroxysteroid dehydrogenase^16–24^. For other bacteria, individually mineralizing estrogen is difficult in the absence of one or more of these enzymes. On the other hand, exogenous microorganisms have difficulties in surviving in new conditions due to competition; however, the synergistic effect among microorganisms enhances the function and ability for environmental adaptation^25^. Isabelle *et al*. isolated an estrogen-degrading bacterial consortium containing five types of bacteria from bio-treated swine waste water; this consortium could remove synthesized equol-D4 in cultural media. However, none of the five strains alone could degrade the synthesized equol-D4^26^. Under these circumstances, using the synergistic effect between indigenous bacteria is one of the best strategies to degrade estrogens in wastes.

In China, animal manures such as poultry and livestock wastes are mostly used in land applications. Accordingly, estrogens have been discharged into the aquatic environment via surface runoff or land-applied compost, thereby posing risks to organisms in various areas^7,27^.

In light of the rich estrogen-degrading bacteria isolated from waste water, activated sludge, the sea, poultry and livestock wastes, and other environmental media, we hypothesized that (1) bacteria that could rapidly degrade 17β-estradiol by the synergistic effect are present in livestock and poultry wastes and that (2) the synergistic effect among bacteria enhances their adaptability to the environment. In the present study, two bacterial strains that exhibit a synergistic effect were isolated and identified. Furthermore, the effect of many factors such as nitrogen source, C/N ratio, pH, carbon coexistence, and heavy metals on the bacterial growth and 17β-estradiol degradation were also studied.

## Results and Discussion

### Isolation of 17β-estradiol-degrading bacterial co-culture

**Figure 1:**
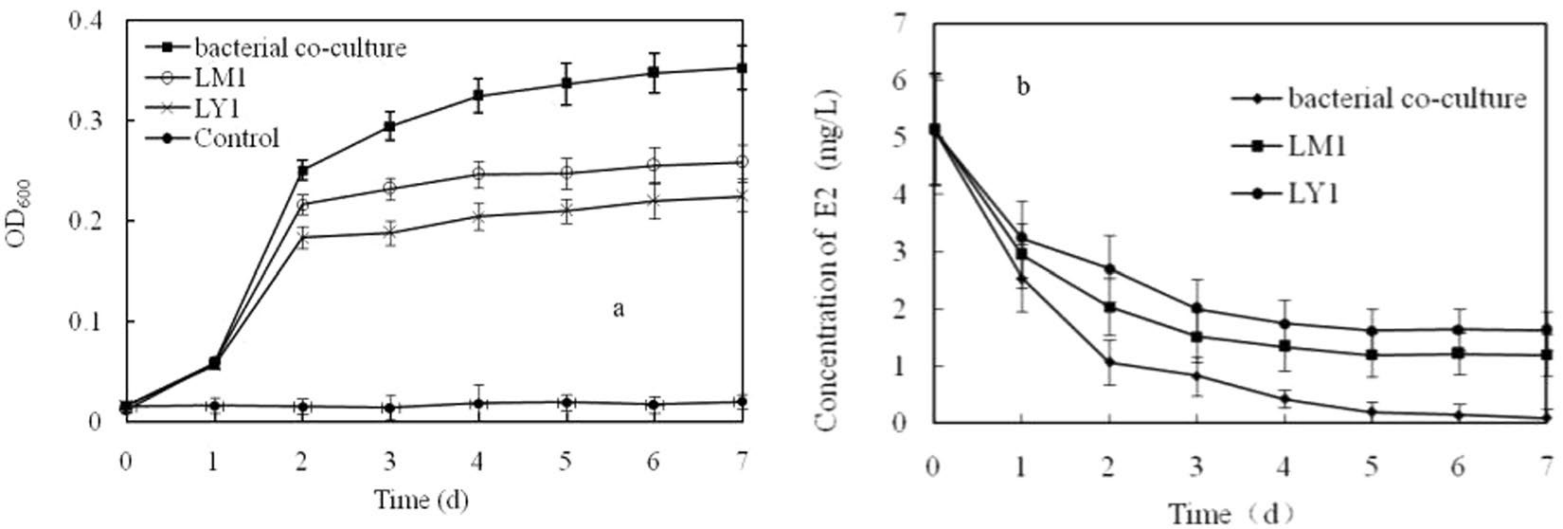
Growth of LM1, LY1 and bacterial co-culture containing the strains LM1 and LY1 (**a**) and the corresponding E2 degradation (**b**) in acetone-free mineral salt medium with 5 mg L^−1^ of E2 during 7 days of incubation in a shaker (150 rpm) at 25 °C in the dark.

Two strains of bacteria with a strong ability to degrade E2 were successfully isolated from the composite samples after 50 days of enrichment cultivation. They were arbitrarily named as strains LM1 and LY1. Figure 1a shows that the bacterial co-culture containing strains LM1 and LY1 continually grew with an average maximum OD_600_ of 0.352 during the 7 days of incubation. In comparison, after 2 days of incubation, the individual growth of strains LM1 and LY1 almost stopped and the average maximum OD_600_ was 0.228 and 0.197, respectively. The bacterial co-culture containing LM1 and LY1 bacterial strains could rapidly remove approximately 98% E2 when 5 mg L^−1^ of E2 was used as a sole carbon source within 7 days of incubation. However, concentration of E2 decreased only 77% and 68% when strains LM1 and LY1 were incubated alone, respectively (Fig. 1b). Overall, the bacterial co-culture could grow on E2 as the sole carbon source and continually utilize the metabolite, which led to the continuous growth of bacteria and decrease of E2. In the case of individual growth, lethal intermediate metabolites may be produced by strain LM1 or LY1, which led to unchanged bacterial growth and E2 degradation after the logarithmic phase. Yu *et al*. divided the degradation of E2 by a single bacterial strain into three patterns according to the degree of estrogens being transformed and the estrogenicity of metabolites and/or the end products of estrogen degradation^28^. In pattern A, the concentration of estrone increased and accumulated over the experimental period due to the degradation of E2, and no degradation of estrone was observed. In pattern B, the transformation of E2 to estrone occurred much more slowly and only a small part of produced estrone was degraded. In pattern C, degradation of E2 and estrone were much improved with no estrogenic metabolites and/or end products from E2 degradation. In addition, several bacteria reversibly converted E2 to E1 but could not decrease estrogenic activity^26^. According to the E2 biodegradation trend, the bacterial co-culture containing strains LM1 and LY1 may degrade E2 in pattern C. By comparison, the individual degradation of E2 by strain LM1 or LY1 may be recognized as pattern B. So, we can infer that there are different metabolic mechanisms which prevent the further degradation of E2 by strain LM1 or LY1. When strains LM1 and LY1 are together, this inhibitory can be overcome through inter-organism interactions^29^. Nevertheless, the bacterial co-culture may play an important role in the degradation of estrogens from livestock and poultry manure. Therefore, strains LM1 and LY1 were identified, and their ability to degrade E2 under different conditions was tested.

### Characterization of strains LM1 and LY1

The following is the characterization of physiological and biochemical reactions for strain LM1: Gram-negative, rod-shaped, catalase-positive, oxidase-negative and grew on glucose with acidification. In addition, Strain LM1 utilized glucose and lactose. And hydrogen sulfide test showed that there was no hydrogen sulfide produced. Strain LY1 was Gram-negative, short rod-shaped, had no spores, and grew on succinate with alkalization. In addition, strain LY1 utilized citrate and malonate.

**Figure 2:**
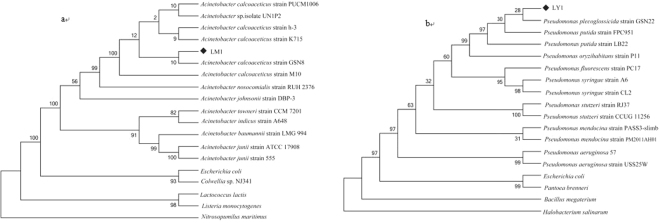
The phylogenetic tree constructed base on 16 S rDNA gene sequences. Bootstrap values calculated from 1000 replications are given at each branching point. ^◆^LM1 and ^◆^LY1: the strains isolated in our research.

The analysis of 16 S rDNA gene sequences of strains LM1 and LY1 revealed that they exhibited the highest similarity to the type strains of *Acinetobacter* and *Pseudomonas*, respectively (Fig. 2a,b). This analysis, along with the morphological and biochemical characteristics of the isolates, suggested that the bacterium LM1 belonged to the genus *Acinetobacter* and is most closely related to *Acinetobacter calcoaceticus* strain M10. Strain LY1 belonged to the genus *Pseudomonas* and is most closely related to *Pseudomonas putida* strain LB22. Isabelle *et al*. isolated one strain of *Pseudomonas* that could convert E2 to estrone from activated sludge bioreactor treating swine waste^20^.

### Degradation kinetics of E2 by bacterial co-culture

**Figure 3:**
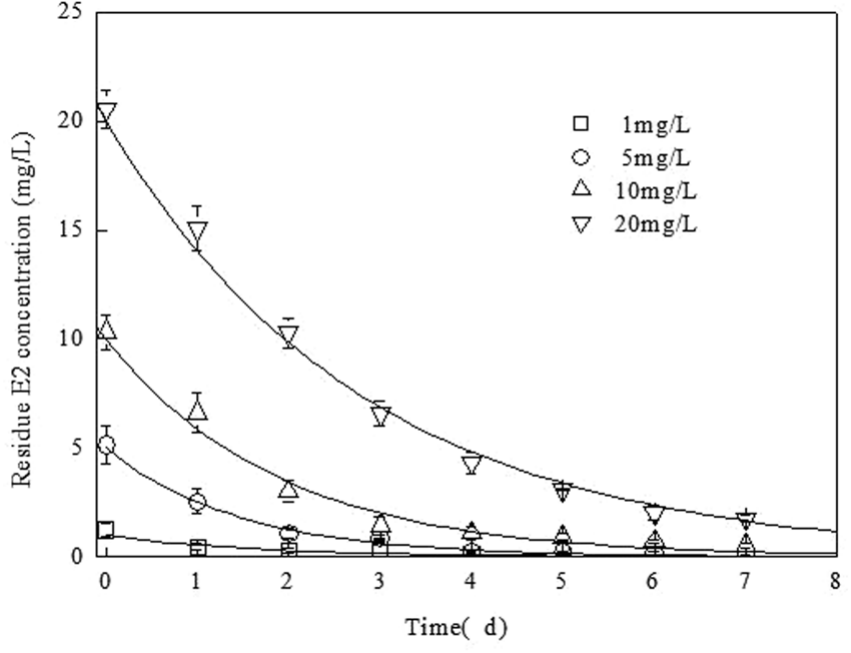
Degradation of E2 by bacterial co-culture containing the strains LM1 and LY1 in acetone-free mineral salt medium with 17β-estradiol at concentrations of 1, 5, 10 and 20 mg L^−1^ during 7 days of incubation in a shaker (150 rpm) at 25 °C in the dark.

The degradation of E2 by bacterial co-culture at concentrations ranging from 1 to 20 ml L^-1^ was investigated. E2 degradation by bacterial combination was fitted by first-order kinetic equation (Fig. 3) as follows:

1 $$C={C}_{0}{e}^{-Kt}.$$

Where *C*_0_ is initial concentration of E2 (mg L^−1^), *C* is E2 concentration at t time (mg L^−1^), t is time (d), *K* is the first order rate constant. The half-life of E2 biodegradation by bacterial combination was calculated according to the equation:

2 $${t}_{1/2}=\,\mathrm{ln}\,2/K.$$

The kinetic equations and the relative parameters for the biodegradation of E2 at different initial concentrations are listed in Table 1. The calculated results showed that E2 biodegradation reaction fitted well with first-order kinetics. When the initial concentration of E2 was increased from 1 to 20 mg L^−1^, the first order rate constant and t_1/2_ changed from 1.004 to 1.136 d and 1.298 to 1.947 d, respectively. The results showed that most E2 at the initial concentration of less than 10 mg L^−1^ was quickly degraded within 3 days of incubation. After 7 days of incubation, the degradation extent was 99%, 98% and 95% when the initial concentration of E2 was 1, 5 and 10 mg L^−1^, respectively. More time was needed for bacterial co-culture to accommodate, grow and degrade E2 when E2 concentration was increased to 20 mg L^−1^. However, the bacterial co-culture still degraded 91% of E2 after 7 days of incubation. These results show that E2 would be quickly removed by bacterial co-culture in application because the concentration of estrogens in livestock and poultry wastes is very low^5,7^. The ability of bacterial co-culture to degrade E2 was not the strongest among the E2-degrading bacteria isolated from different sources^3,11,12^. Nevertheless, bacterial co-culture has significant practical value because the strains LM1 and LY1 were indigenous bacteria and their synergistic action may improve their survivability in actual microenvironment.

**Table 1:** First order kinetics parameters of E2 degradation by bacterial co-culture.

Initial concentration of E2 (mg L^−1^)	*K*	*R* ^2^	*t*_*1/2*_(d)
1	0.690 ± 0.029	0.995	1.004
5	0.610 ± 0.093	0.942	1.136
10	0.534 ± 0.036	0.984	1.298
20	0.356 ± 0.012	0.993	1.947

### Effect of nitrogen source and C/N ratio on bacterial growth and E2 degradation

**Figure 4:**
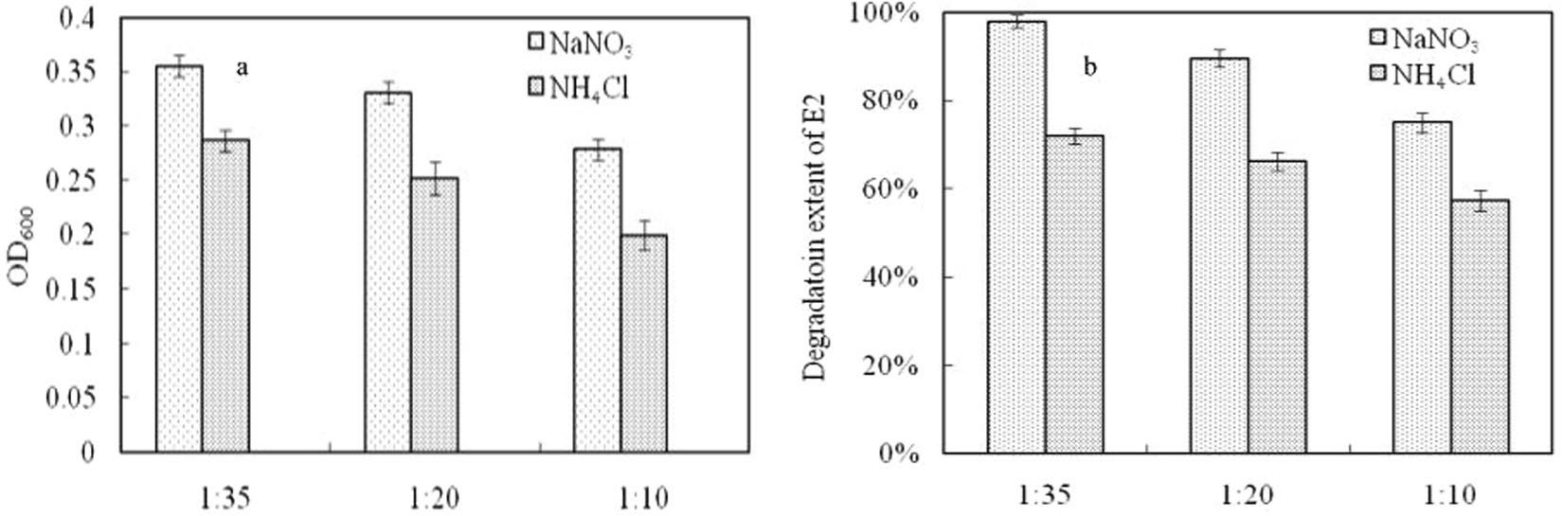
Effect of nitrogen source and C/N ratio on bacterial co-culture growth (**a**) and E2 degradation (**b**).

Two types of nitrogen sources (NH_4_^+^ and NO_3_^−^) were used to test the effect of nitrogen source on bacterial growth and E2 degradation. As shown in Fig. 4(a,b), the bacterial co-culture could use two kinds of nitrogen (NH_4_^+^ and NO_3_^−^) to grow and metabolize E2. However, at the same C/N ratio, the bacterial co-culture grew more efficiently when NO_3_^−^ was used as the sole nitrogen source instead of NH_4_^+^. The reason may be different metabolic and growth pathways when the form of N was changed, for example the nitrification or denitrification. For example, the degradation extent of E2 was 98% and 72% when NO_3_^−^ or NH_4_^+^ was used as a sole nitrogen source at a C/N ratio of 1:35. It was found that most E2 degrading bacteria were isolated through a medium culture in which NH_4_^+^ was used as the sole nitrogen source^12,13,26^. However, Yu *et al*. isolated 14 strains of E2 degrading bacteria from the activated sludge in a waste water treatment plant through a nitrate salt containing culture medium^28^. Kurisu *et al*. isolated two strains of E2-degrading bacteria from soil samples through an enrichment culture in a medium containing NH_4_Cl and NaNO_3_^4^. So, nitrogen form used in the mineral salt medium maybe significantly affect bacterial growth and E2 degradation and further research is needed to investigate the underlying mechanisms. Generally, NH_4_^+^ and NO_3_^−^ simultaneously exist in poultry and livestock wastes and polluted environments. Therefore, bacteria that can utilize either NO_3_^−^ or NH_4_^+^ would have important practical application value.

From Fig. 4b, it can be seen that degradation extent of E2 by bacterial co-culture decreased with increasing C/N ratio. For example, the degradation extent of E2 decreased from 98% to 75% when the C/N ratio increased from 1:35 to 1:10 in a culture medium with NO_3_^−^ as the sole nitrogen source. The above results show that high nitrogen content in culture mediums promotes bacterial growth and consequently contributes to E2 degradation. So, the C/N ratio may be an important factor that affects the effectiveness of E2 degradation by bacteria because the C/N ratio of most of the wastes and environmental samples is greater than 5:1^30^.

### Effect of pH on E2 degradation

**Figure 5:**
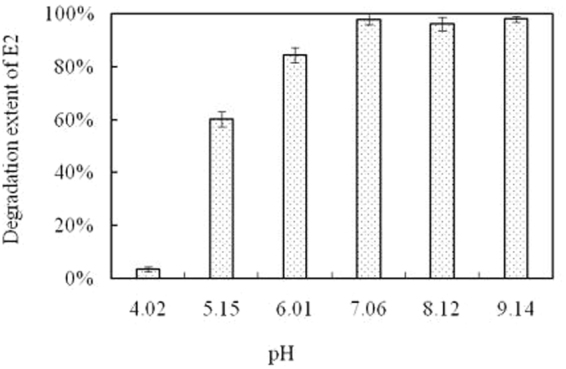
Effect of pH on E2 degradation by bacterial co-culture. pH of the mineral salt medium was adjusted to 4.02, 5.15, 6.01, 7.06, 8.12 and 9.14 using 0.1 mol L^−1^ NaOH or 0.1 mol L^−1^ HCl.

The effects of the culture medium pH on bacterial growth and E2 degradation are shown in Fig. 5. The E2 degradation extent increased when pH increased from 5.15 to 7.06. The almost quantivalent E2 degradation extent was for pH 7.06, 8.12 and 9.14. Degradation of E2 by bacterial co-culture was almost inhibited in the relatively acidic (pH 4.02 and 5.15) condition. However, 98% of E2 was degraded even at pH 9.14. As a whole, the pH at which bacterial co-culture satisfactorily degraded E2 ranged from 7.00 to 9.00, which is meaningful for their application in the bioremediation of estrogen-polluted environments.

### Effect of coexisting carbon source on E2 degradation

**Figure 6:**
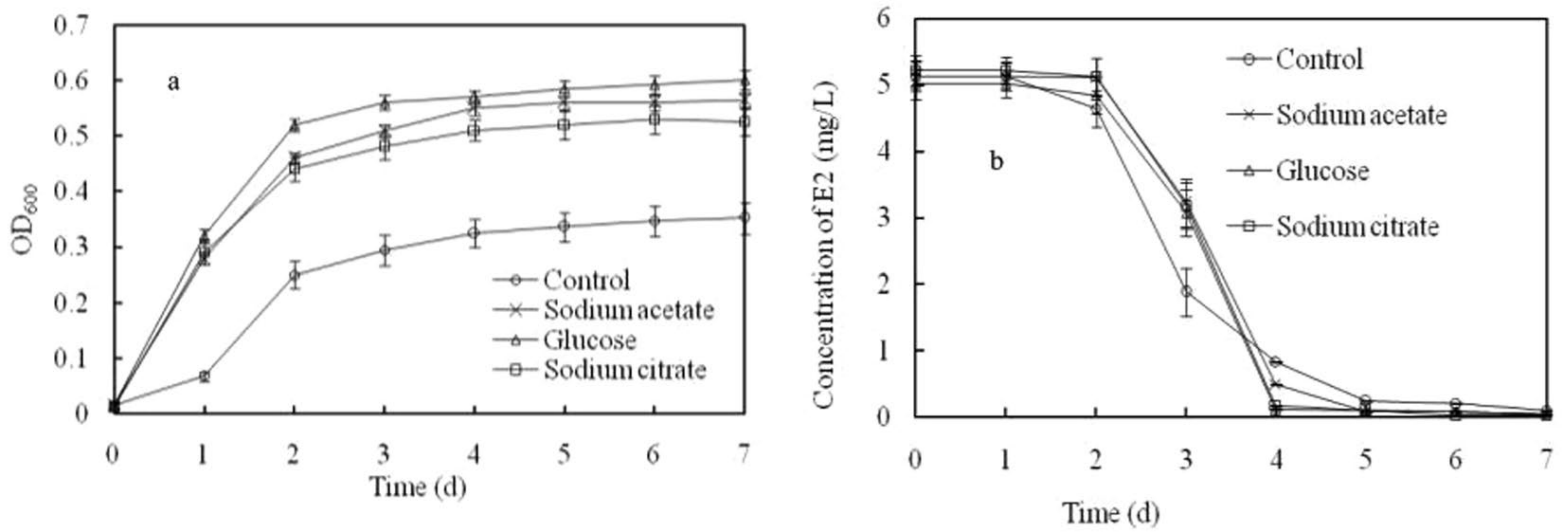
Effect of carbon coexistence with E2 on bacterial co-culture growth (**a**) and E2 degradation (**b**). The carbon sources coexisting with E2 were sodium acetate, glucose and sodium citrate. The carbon content ratio of E2 to other carbons was 1:1.

As shown in Fig. 6(a,b), adding other carbon sources (sodium acetate, glucose and, sodium citrate) to the acetone-free MS medium with 5 mg L^−1^ of E2 promoted the growth of bacterial co-culture, thereby showing that bacterial co-culture can grow on the three carbon sources. E2 was degraded by 63.4% when E2 was used as the sole carbon source in the first 3 days of incubation, whereas the E2 degradation extent was only approximately 37.2%, 38.7% and 38.9%, respectively, when sodium acetate, glucose, sodium citrate were added to the culture medium for the first 3 days of incubation. However, E2 was rapidly degraded from 3 to 4 days in the carbon source coexistence cultural medium. Consequently, the E2 degradation extent was slightly higher in carbon coexistence culture medium than in the sole carbon culture medium after 4 days of incubation. These data showed that the addition of other carbon source affected the E2 degradation by bacterial co-culture, but the bacterial growth was promoted and thus increased the degradation extent of E2 after the other carbon source was depleted.

### Effect of heavy metals on bacterial growth

**Figure 7:**
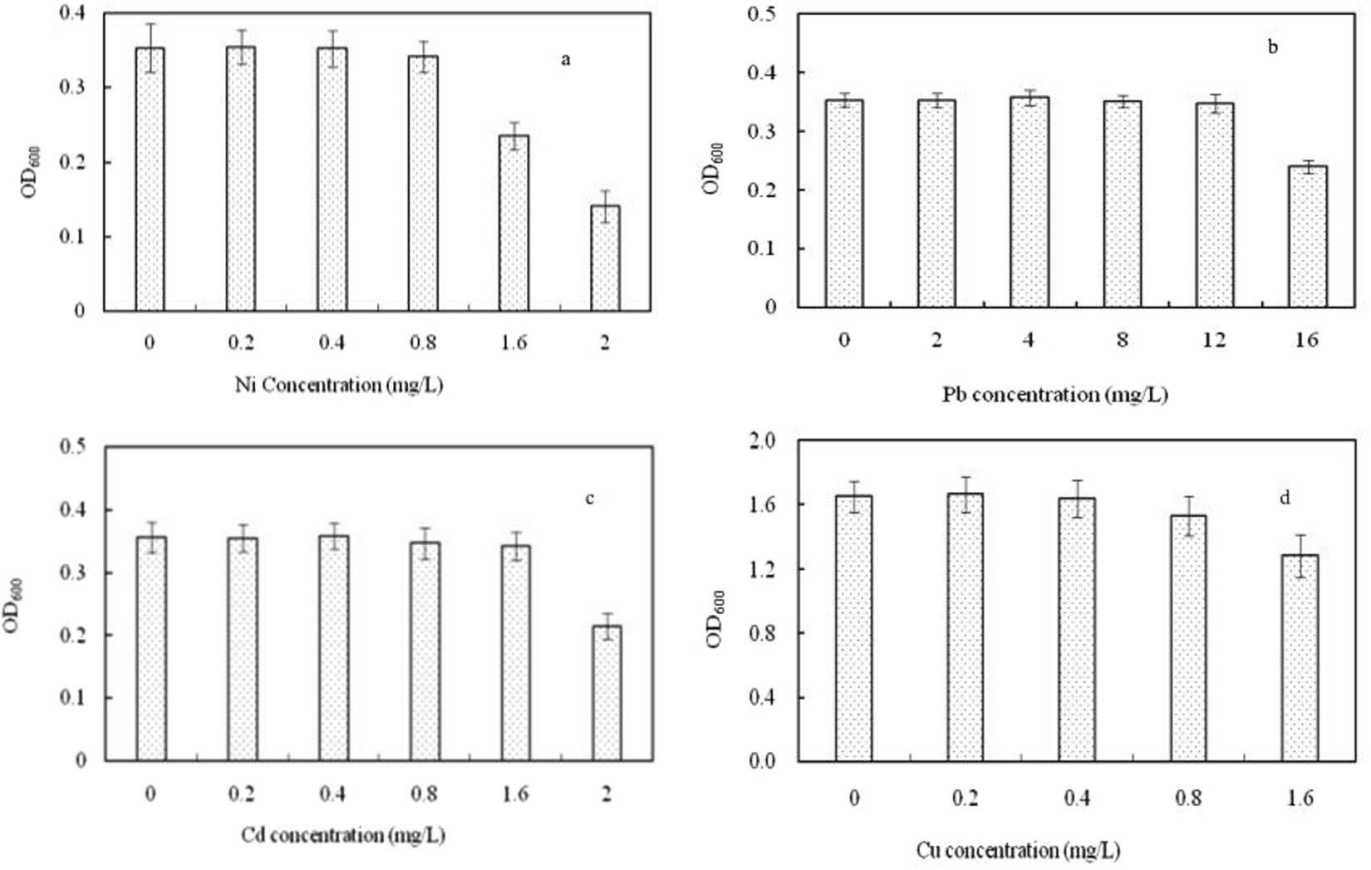
Effect of heavy metals on bacterial co-culture growth. Four types of heavy metals, such as Ni (**a**), Pb (**b**), Cd (**c**) and Cu (**d**), at different concentrations were added to the culture medium. The concentration was set according to the environmental standards.

Heavy metal Cu is frequently detected because it can promote animal growth and disease resistance and has been used as feed additives in China. Cd, Pb and Ni are usually found in environment due to the release of pollutants and the application of phosphate fertilizer^31^. Thus, the effect of Cu, Ni, Pb and Cd on bacterial growth was investigated (Fig. 7). The growth of the bacterial co-culture at the initial stage (after 3 days of incubation) was adversely affected when the concentrations of Ni, Pb, Cd and Cu were all 1.6 mg L^−1^, respectively. The growth of the bacterial co-culture was not significantly decreased by Ni, Pb, Cd or Cu at or below 0.8 12, 1.6 or 0.8 mg L^−1^. Notably, the bacterial co-culture was more resistant to Pb than other heavy metals. These results also show that application of a bacterial co-culture would not be affected by heavy metals in the environment because the available ions of Cu, Ni, Pb and Cd were lower than the highest concentration used in the experiments.

## Conclusion

Two Gram-negative strains (LM1 and LY1) isolated from livestock and poultry wastes were identified as 17β-estradiol-degrading bacteria. An analysis of 16 S rDNA gene sequences identified them as belonging to the genus *Acinetobacter* (LM1) and *Pseudomonas* (LY1). The bacterial co-culture containing LM1 and LY1 bacterial strains could rapidly remove approximately 98% of E2 when 5 mg L-1 of E2 was used as a sole carbon source within 7 days of incubation. However, E2 was degraded by 77% and 68% when strains LM1 and LY1 were incubated alone, respectively. More than 90% of E2 could be removed by bacterial co-culture when E2 concentration was equal or less than 20 mg L^−1^. The bacterial co-culture utilized two types of nitrogen sources (nitrate > ammonium) to support the growth and degrade E2. Low C/N ratio was more suitable for bacterial growth and E2 degradation. The optimal pH level for bacterial co-culture to degrade E2 ranged from 7 to 9. The coexisting sodium acetate, glucose, or sodium affected E2 degradation by bacterial co-culture in the first 4 days, but more E2 was removed by bacterial co-culture when they were depleted. The growth of the bacterial co-culture was not significantly decreased by Ni, Pb, Cd or Cu at or below 0.8, 12, 1.6 or 0.8 mg L^−1^. In view of the results here obtained, the two strains possess potential for their application in the biodegradation of animal wastes. To reveal the mechanism of degradation, the metabolites produced by bacterial co-culture should be identified, and the enzymes involved in the estrogen biodegradation, such as dehydrogenase and carbonyl enzymes, should be further studied in detail.

## Methods

### Chemicals

E2 (>99% pure), purchased from Sigma–Aldrich and dissolved at a concentration of 1000 mg L^−1^ in acetone, were used as stock solutions. The organic solvents, methanol, ethyl acetate, acetonitrile, and HPLC-grade acetone were also obtained from Sigma–Aldrich. All other chemicals were of the highest commercially available purity.

### Enrichment and isolation of 17β-estradiol-degrading bacteria

Waste samples (including poultry, livestock wastes, manure compost, and manure-applied soil collected from Jilin Province, Northeast China) were used as inoculum source for obtaining the 17β-estradiol-degrading bacteria. To ensure the diversity of bacteria in the inocula, a composite sample was prepared by mixing 40 individual samples (1:1, mass ratio). Bacterial suspension was prepared by adding 5 g of the composite sample into a 100-ml flask containing 50 ml of sterilized water. The suspension was stirred by hand for 30 s and was left to stand for 20 min thereafter. A 10-ml aliquot of the resulting bacterial suspension was added to 100-ml acetone-free mineral salts (MS) medium with 5 mg L^−1^ of E2 as the sole carbon source. The suspension was then incubated at 150 rpm and 25 °C in the dark until the cultures became turbid and the E2 concentration significantly decreased (after 5 days of incubation). Then 1 ml of the enrichment culture was transferred into 100 ml of fresh acetone-free MS medium with 5 mg L^−1^ of E2 and re-incubated under the same condition for 5 days. This procedure was repeated five times. The acetone-free MS medium was prepared as follows: known amounts of estrogen stock solution were added to the MS medium, the medium was heated to 80 °C, and the medium was then purged with air at a flow rate of 120 ml min^−1^ for 30 min to remove the remaining acetone from the medium. After cooling to room temperature, the acetone-free MS medium was used for degradation experiments^28^. The MS medium comprised (per liter of distilled water) 1.0 g of K_2_HPO_4_, 0.6 g of NaH_2_PO_4_·2H_2_O, 0.2 g of MgSO_4_·7H_2_O, 0.2 g of KCl, 1 g of NaNO_3_, and 1 ml of trace element solution, as described by Park *et al*.^25^.

After 50 days of enrichment incubation, the last enrichment culture was used as a bacterial source for the isolation of 17β-estradiol-degrading bacteria. One milliliter of the culture was serially diluted and plated on R2A agar plates containing 5 mg L^−1^ of E2. The plates were incubated at 25 °C in the dark for 24–48 h. The morphologically distinct colonies were selected and purified by streaking on R2A agar plates. The 17β-estradiol-degrading experiment was performed by incubating strain LM1, LY1 or their co-culture in acetone-free MS medium with 5 mg L^−1^ of E2 as a sole carbon source. Finally, the bacterial co-culture consisting of strains LM1 and LY1 (arbitrarily named), which showed a superior degradation towards to E2, were used for further study.

### Characterization of strains LY1 and LM1

Cells of strain LM1 or LY1 were suspended in sterilized water and boiled at 100 °C for 5 min. One milliliter of the above lysate as a template was used to amplify the 16 S rDNA gene sequences of strain LM1 or LY1 via polymerase chain reaction (PCR). The universal eubacterial primers Pf (5′-agagtttgatcctggctcag-3′) and Pf (5′-acggctaccttgttacgact-3′), which represent bp 8–27 and 1495–1514 of the *Escherichia coli* 16 S rDNA (accession no. E05133), were used as forward and reverse primers, respectively. The PCR mixtures (50 μl) contained 5 μl of 10 × Ex Taq Buffer (containing Mg^2+^), 1 μl of Ex Taq enzyme (TaKaRa), 5 μl of dNTP mixture, 5 μl of each primer, and 5 μl of DNA template, adjusted to 50 μl with sterile ddH_2_O. PCR amplification was performed using a Gene Amp PCR system 9600 thermocycler (Perkin-Elmer, Norwalk, CT, USA) programmed as follows: 5 min of denaturation at 94 °C; 30 cycles of 94 °C for 30 s, 55 °C for 30 s, and 72 °C for 90 s; final extension at 72 °C for 10 min. The amplified PCR products were excised from 1.2% (w/v) agarose gel and ligated into the pGEM-T easy vector (Promega, Madison, WI, USA) according to the manufacturer’s instructions. The 16 S rDNA sequence was determined by Jilin Kumei Biotech Company (China). A BLAST similarity search of the nucleotide sequence retrieved sequences from species within the genus *Acinetobacter* and *Pseudomonas*. A multiple alignment of all the sequences included in the analysis was produced by ClustalX 2.1. The region of this alignment, in which all the sequences overlap (with intermittent gaps and uncertain bases omitted) was used to construct a phylogenetic tree using the neighbor-joining method in MEGA 6.0^32^.

In addition, morphological appearance, Gram-staining results, and other physiological and biochemical characteristics were used to identify the isolated bacteria. The two strains were deposited in the China General Microbiological Culture Collection Center in May 18, 2015. The CGMCC numbers of strains LM1 and LY1 are 10814 and 10815, respectively.

### Preparation of bacterial co-culture cell suspension

A single colony of strain LM1 or LY1 was grown in 10 ml of acetone-free MS medium containing 5 mg L^−1^ of E2 at 150 rpm and 25 °C. After 5 days of cultivation, the cultures were wholly transferred to 100 ml of the above fresh medium. They were then incubated under the same conditions for another 5 days. Then the culture was centrifuged at 8000 rpm for 5 min to collect the bacterial cells. The harvested cells of strains LM1 and LY1 were washed three times with MS medium and finally suspended in fresh MS medium at an OD_600_ of 0.1. To obtain bacterial co-culture cell suspensions, the two cell suspensions were mixed at a volume ratio of 1:1 because the results from the viable cell count experiment showed that the cell quantity ratio of strain LM1 to LY1 in the cultures was approximately 1:1.

### Degradation of E2 at different concentrations by bacterial co-culture

In this experiment, 250-ml Erlenmeyer flasks containing acetone-free MS medium with different initial concentrations of E2 and 2% of cell suspension were incubated in a rotary shaker (150 rpm) at 25 °C in the dark. The initial concentrations of E2 were set as 1, 5, 10 and 20 mg L^−1^. At designated intervals, culture samples were collected for the determination of OD_600_ and E2 residue.

### Effect of factors on bacterial growth and E2 degradation

The experiment on the effect of nitrogen source on bacterial growth and 17β-estradiol degradation was performed by using 1.0 g L^−1^ of NaNO_3_ or 0.63 g L^−1^ of NH_4_Cl as a sole nitrogen source in the acetone-free MS medium. To investigate the effect of molar concentration ratio of carbon to nitrogen (C/N ratio) on bacterial growth and E2 degradation, the C/N ratios were set as 1:35, 1:20 and 1:10 by changing the amount of nitrogen source in the culture medium with 5 mg L^−1^ of E2, and we adjusted the pH by adding solution of HCl or NaOH to be 7.0. Regarding the pH effect, MS media with six different pH values (4.02, 5.15, 6.01, 7.06, 8.12 and 9.14) adjusted with 0.1 mol L^−1^ HCl and 0.1 mol L^−1^ NaOH were used. Experiments on the effect of carbon coexistence on bacterial growth and 17β-estradiol degradation were conducted by adding 22.5 mg L^−1^ of sodium acetate trihydrate, 10.9 mg L^−1^ of glucose and 14.2 mg L^−1^ of sodium citrate to the acetone-free MS medium with 5 mg L^−1^ of E2. The effect of heavy metals on bacterial growth were evaluated by adding Ni (0, 0.2, 0.4, 0.8, 1.6 and 2 mg L^−1^), Pb (0, 2, 4, 8, 12 and16 mg L^−1^), Cd (0, 0.2, 0.4, 0.8, 1.6 and 2 mg L^−1^), and Cu (0, 0.2, 0.4, 0.8 and 1.6 mg L^−1^) to the MS medium in form of metal nitrate. After 5 days of incubation, samples were collected to measure OD_600_.

The above E2 degradation experiments were conducted in a series of 250-ml flasks containing 100 ml of the specific cultural medium, 2% of cell suspension, and 5 mg L^−1^ of E2. The flasks were incubated in a shaker (150 rpm) at 25 °C in the dark. As a control without inoculation, 50 ml acetone-free MS medium with 5 mg L^−1^ of E2 was submitted to the same experimental conditions. All the above experiments were conducted in triplicate, and the data were expressed as means ± standard deviations.

### Estrogen analysis

Bacterial cells were separated from the cultures by centrifuging them at 10,000 rpm for 10 min at 4 °C. Estrogen in the supernatant was extracted with ethyl acetate and concentrated by evaporating ethyl acetate and re-dissolving the residual precipitates in methanol. The final solution was filtered through a 0.22-µm membrane and analyzed using a high-performance liquid chromatography (Agilent 1260) system equipped with a UV detector (*λ* = 205 nm), a 3-cm C-18 guard column, and a Lua end-capped (C18) reversed-phased column (4.6 × 150 mm, 5 µm particles; Phenomenex, Torrance, CA). 50-µl injection volumes were used; the mobile phase comprised acetonitrile and water at a volume ratio of 45:55 and had a flow rate of 1.0 ml min^−1 ^^14,33^. The column temperature was adjusted to 25 °C. Retention time for E2 was 7.28–7.45 min. External calibration curves were generated to estimate sample concentrations from peak areas and were linear between 10 µg L^−1^ and 10 mg L^−1^. In addition, an internal standard was set to correct for losses during processing.
